# Eco-anxiety: A new challenge for clinical psychiatry

**DOI:** 10.1192/j.eurpsy.2026.10992

**Published:** 2026-07-31

**Authors:** I. M. Marques

**Affiliations:** Department of Psychiatry, Unidade Local de Saúde de Coimbra, Coimbra, Portugal

## Abstract

**Introduction:**

Climate change is a highly relevant issue. Eco-anxiety is a complex construct that refers to concern or distress related to climate change and its implications for the future. Feelings of hopelessness and helplessness regarding this issue can exacerbate eco-anxiety, partly due to increased public awareness.

**Objectives:**

The aim is to provide a review of the concept of eco-anxiety and to discuss its challenges for clinical practice.

**Methods:**

Literature review on the subject, using databases such as PubMed.

**Results:**

Climate change resulting from global warming is currently affecting both the environment and public health. It can influence mental health directly, indirectly, and through awareness of the issue (Heinz A, Brandt L. Lancet Reg Health Eur 2024; 43:100969). In terms of awareness, this has been increasing due to media coverage. Various studies show that the vast majority of individuals, regardless of age, express concern about environmental issues that affect daily functioning (Baudon & Jachens. Int J Environ Res Public Health 2021; 18:9636). The term eco-anxiety encompasses not only anxiety but also a spectrum of emotions, including fear, anger, exhaustion, and despair. Eco-anxiety can also be understood as an adaptive response to climate threats, with the potential to promote pro-environmental behavior (Betro S. Front Psychiatry 2024; 15:1429571). Focusing on meaning may be important, as it can encourage individuals to trust their beliefs and values and to act adaptively during periods of stress (Betro S. Front Psychiatry 2024; 15:1429571). This may serve as an advantageous coping strategy in the context of climate change. Studies suggest that approaches such as psychoanalysis, analytical psychology, acceptance and commitment therapy (ACT), and mindfulness techniques can provide useful strategies for managing symptoms associated with eco-anxiety (Elliston. Australasian Psychiatry 2024; 32:97). The level of evidence, however, varies, with ACT and mindfulness-based interventions showing the most promising results to date. Dialectical behavior therapy (DBT) may also be helpful in managing catastrophic thoughts related to climate distress by using emotional regulation skills (Elliston. Australasian Psychiatry 2024; 32:97). The starting point for any intervention should be empathetic and non-judgmental listening, rather than reassurance. Currently, several eco-anxiety questionnaires have been developed to assess its various dimensions.

**Conclusions:**

Climate change is one of the greatest health threats of the 21st century. Given its relevance and urgency, more training is needed to equip healthcare professionals with the skills to apply in practice, based on a holistic model. Finally, it is important to emphasize that the goal is not to pathologize eco-anxiety, but rather to understand it as a spectrum.

**Disclosure of Interest:**

None Declared

